# PINCH-1 promotes tumor growth and metastasis by enhancing DRP1-mediated mitochondrial fission in head and neck squamous cell carcinoma

**DOI:** 10.1080/15384047.2025.2477365

**Published:** 2025-03-11

**Authors:** Ruxian Tian, Hao Song, Jiaxuan Li, Ting Yuan, Jiahui Liu, Yaqi Wang, Yumei Li, Xicheng Song

**Affiliations:** a Department of Otorhinolaryngology, Head and Neck Surgery, Yantai Yuhuangding Hospital, Qingdao University, Yantai, Shandong, China; b Shandong Provincial Clinical Research Center for Otorhinolaryngologic Diseases, Yantai, Shandong, China; c Shandong Provincial Key Laboratory of Neuroimmune Interaction and Regulation, Yantai, Shandong, China; d Yantai Key Laboratory of Otorhinolaryngologic Diseases, Yantai, Shandong, China

**Keywords:** DRP1, growth, HNSCC, metastasis, PINCH-1

## Abstract

**Purpose:**

Abnormal expression of PINCH-1 has been observed in various types of human cancers. However, the clinical importance and mechanism underlying its role in head and neck squamous cell carcinoma (HNSCC) is yet to be fully elucidated.

**Methods:**

This study evaluated the expression of PINCH-1 in HNSCC samples through immunohistochemical staining and Western blotting. AMC-HN-8, Cal27, and SCC7 cell lines were utilized for cellular function experiments, both in vivo and in vitro. CCK8, colony-formation assay, flow cytometry, wound-healing assay, and transwell assay were employed to investigate the effects of alterations in target proteins on the growth and metastasis of cancer cells. Mito-Tracker Deep Red FM was used to track mitochondrial morphology.

**Results:**

PINCH-1 was found to be overexpressed in HNSCC and closely associated with lymph node metastasis and poor pathologic differentiation. Its upregulation promoted proliferation, inhibited apoptosis, and enhanced migration and invasion in HNSCC cells. It also promoted mitochondrial fission. We conducted a mechanism analysis, which showed that PINCH-1 knockdown inhibited mitochondrial fission by reducing the expression of DRP1. Furthermore, inhibition of mitochondrial fission could impede the proliferation and metastasis of HNSCC cells. Re-expression of DRP1 reversed the inhibitory effect of PINCH-1 knockdown on mitochondrial fission, cell proliferation, and metastasis in HNSCC cells.

**Conclusions:**

PINCH-1 plays a critical oncogenic role in HNSCC by enhancing DRP1-mediated mitochondrial fission, which may serve as a novel therapeutic target for HNSCC.

## Introduction

Head and neck squamous cell carcinoma (HNSCC) originates from the mucosal epithelium of the oral cavity, pharynx, and larynx, and is the most prevalent malignant neoplasm in this anatomical region.^1,2^ Although the therapeutic strategies for HNSCC have seen substantial improvements over the past decade, no significant improvement has been made in patient survival, particularly among those experiencing recurrence or metastasis.^3,4^ Therefore, it is crucial to investigate the underlying molecular mechanisms driving tumorigenesis and metastasis in HNSCC in order to identify more effective therapeutic targets and improve the prognosis.

Emerging evidence has shown that alterations in mitochondrial dynamics play a positive role in various aspects of tumor initiation and progression.^5^ Mitochondria are dynamic organelles that constantly undergo fusion and fission processes, known as mitochondrial dynamics, to meet the requirements of cells.^6^ The dynamic balance between mitochondrial fusion and fission is essential for regulating various processes, including mitochondrial quality control, energy metabolism, cell proliferation, and cell migration.^7^ Imbalanced mitochondrial dynamics are believed to be implicated in cancer development.^8,9^ Excessive mitochondrial fission can increase the malignancy of cancer cells.^10–12^ For example, high mitochondrial fission enhances the migratory and invasive abilities of lung cancer cells.^13^ Furthermore, excessive mitochondrial fission can increase the resistance of tumor cells to chemotherapy.^14^ Dynamin-related protein 1 (DRP1) plays a key role in regulating mitochondrial fission. DRP1 is recruited from the cytoplasm to the outer mitochondrial membrane to promote mitochondrial fission.^15^ The upregulation of DRP1 has been observed in pancreatic cancer, lung cancer, colorectal cancer, and esophageal cancer.^16–19^ Among them, Liang et al. found that DRP1-mediated mitochondrial fission promotes the growth and metastasis of pancreatic cancer by enhancing aerobic glycolysis.^16^ Glycolysis can rapidly provide energy, which is beneficial for tumor cells to maintain their rapid proliferation state and survive better under the hypoxic conditions of the tumor microenvironment.^20^ Moreover, a recent study showed that DRP1 is upregulated in HNSCC tissues and can promote tumor progression and glycolysis.^21^ However, Huang et al. did not investigate whether the oncogenic role of DRP1 is related to mitochondrial fission. In addition, the regulatory mechanism for DRP1 expression is yet to be explored. Further investigations are needed to elucidate these aspects.

Previous studies have indicated that cell – extracellular matrix (ECM) adhesion is related to the regulation of mitochondrial dynamics.^22^ Cell – ECM adhesion plays a major role in regulating the proliferation, apoptosis, migration, and invasion of tumor cells.^23^ The main regulatory factors mediating cell – ECM adhesion are the cell surface adhesion receptors of the integrin family and other components of the integrin adhesome.^24^ Among these regulators, particularly interesting new cysteine-histidine rich protein 1 (PINCH-1) is a widely expressed and evolutionally conserved component of the integrin adhesome.^25^ As an adapter protein comprising five LIM domains and tandem nuclear localization signals, PINCH-1 binds to integrin-linked kinases (ILK) and parvin to form a ternary protein complex critical for controlling cell – ECM adhesion-mediated cell behavior.^26^ However, it is not yet clear whether PINCH-1 is involved in the regulation of mitochondrial dynamics through cell – ECM adhesion. In addition, previous studies have shown that PINCH-1 is overexpressed in many tumor tissues and is also involved in promoting tumor initiation and progression.^27–30^ However, the role and functional mechanism of PINCH-1 in the onset and development of HNSCC need further exploration.

This study analyzes the expression and function of PINCH-1 in HNSCC and its regulatory relationship with mitochondrial dynamics. Our data suggest that PINCH-1 is highly expressed in HNSCC tissues and is associated with lymph node metastasis and poor pathologic differentiation. Furthermore, our in vitro and in vivo experimental results demonstrate that PINCH-1 promotes the growth and metastasis of HNSCC by facilitating DRP1-mediated mitochondrial fission. These findings may help design a novel strategy for therapeutic interventions for HNSCC.

## Material and methods

### Cell lines and tissue samples

Two human HNSCC cell lines (AMC-HN-8 and Cal27) and one murine HNSCC line (SCC7) were acquired from the National Collection of Authenticated Cell Cultures (Shanghai, China). All cells were cultured in Dulbecco’s modified eagle medium (VivaCell, Germany) supplemented with 10% fetal bovine serum (FBS, Gibco, USA) and 1% penicillin – streptomycin solution (Solarbio, China) in an environment containing 5% CO_2_ at 37°C. The working concentration of the mitochondrial division inhibitor (Mdivi-1, Beyotime, China) was 20 μM. A total of 48 hNSCC tissues and 18 para-cancer tissues were collected from Yuhuangding Hospital, from January 6 to October 30, 2022, for immunohistochemical analysis. An additional 12 pairs of HNSCC and para-cancer tissues were collected for Western blotting analysis. All participants provided written, informed consent. This study was approved by the ethics committee of the Yuhuangding Hospital (No. 2024–616).

### Vectors and cell transfections

A lentivirus vector (pSLenti-U6-shRNA-CMV-F2A-Puro-WPRE) containing short-hairpin RNAs (shRNAs) to knock down PINCH-1 (sh-PINCH-1) was generated. ShRNAs targeting human PINCH-1 or scrambled shRNA sequences were generated using the following sequences: sh-PINCH-1: 5′-AAGG TGATGTGGTCTCTGCTC-3′; sh-negative control: 5′-ACGCATGCATGCTTGCTTT-3′. Additionally, lentiviral overexpression vectors for PINCH-1 and DRP1 were constructed by cloning the target genes into pSLenti-CMV-3×FLAG-PGK-Puro-WPRE for target gene overexpression. The vector construction and lentivirus packaging for all target genes and negative controls were carried out by OBiO Technology (Shanghai, China). Lentiviral transfection was conducted in a six-well plate with a cell density of 50%, a medium volume of 2 mL, and a final lentiviral concentration of 1 × 10^6^ TU/mL. After transfection, purinomycin (2 μg/mL) was used to screen for cells that exhibited stable knockdown or overexpression of the target gene.

### Western blotting

The protein sample (30 µg/well) was first separated by using SDS – PAGE gel and then transferred to a poly (vinylidene fluoride) (PVDF) membrane using a Trans-Blot Transfer Slot. The PVDF membrane was incubated with primary antibodies at 4°C overnight. Subsequently, the membrane was incubated with goat anti-rabbit IgG HRP antibodies (1:5000, Affinity, USA) at room temperature for 1 h. The following primary antibodies were used: anti-PINCH-1 (1:1000, Absin, China), anti-DRP1 (1:1000, Absin, China), anti-FIS1 (1:1000, ABclonal, China), anti-MFN1 (1:1000, ABclonal, China), anti-OPA1 (1:1000, Cell Signaling Technology, USA), and anti-Tubulin (1:1000, Affinity, USA). Protein bands were detected using the ChemiScope 6000 imaging system (Clinx Science Instruments, China) using an ECL luminescence reagent (Affinity, USA). The ImageJ software was used for the relative quantitative analysis of protein bands.

### Immunohistochemistry (IHC)

Tissue slides were first heated in an oven at 60°C for 2 h and were then deparaffinized. The slides were further treated by soaking in a citrate antigen retrieval solution (Beyotime, China) at 100°C for 20 min. They were then subjected to a 10-min treatment with 3% hydrogen peroxide and blocked using 5% bovine serum albumin (Shandong Sparkjade Biotechnology, China) at room temperature for 30 min. Next, the anti-PINCH-1 primary antibody (1:100, Absin, China) was applied to the slides and left to incubate overnight at 4°C. Finally, the slides were incubated with goat anti-rabbit IgG HRP antibody (1:200, Affinity, USA) at 37°C for 30 min. The staining process was completed with 3,3′-diaminobenzidine (Beyotime, China) staining and counterstaining with hematoxylin (Beyotime, China). Detailed scoring methods for immunohistochemical staining have been described previously.^31^

### Cell proliferation assay

The cell proliferation capacity was determined using the Cell Counting Kit-8 (Shandong Sparkjade Biotechnology, China) assay. Initially, cells in the exponential growth phase were seeded in 96-well plates at a density of 8000 cells per well with five replicates per group. They were then cultured in an incubator for 12, 24, 36, and 48 h. Next, 10 µL of the CCK8 reagent was added to each well and incubated at 37°C for 90 min. Finally, the absorbance value of each well at 450-nm wavelength was measured using a ThermoMultiskan FC microplate reader (Thermo Fisher Scientific, USA).

### Colony-formation assay

The cells were seeded in six-well plates at a density of 700 cells per well and incubated in incubator for 10 days. Subsequently, the cells were fixed with 4% paraformaldehyde for 20 min and stained with crystal violet for 30 min. They were then washed with phosphate buffer solution and allowed to dry before being photographed for analysis.

### Transwell invasion assays

First, 50 μL of Matrigel (BD Biosciences, NY, USA) diluted in a Dulbecco’s modified eagle medium was added to the upper chamber and incubated at 37°C overnight. The cells were then diluted to 2 × 10^5^/mL using a serum-free medium. Next, 100 µL of this cell suspension was added to the upper chamber. The lower chamber was filled with 700 µL of the medium supplemented with 20% FBS. Next, the cell culture plates were returned to the incubator for 48 h. The upper chambers were cleaned with PBS and fixed with methanol for 30 min. Giemsa stain was subsequently added to the upper chamber for 25 min. Finally, the uninvaded cells in the upper chamber were wiped with a cotton swab, and five fields (200×) were randomly selected under the microscope for photo-recording.

### Wound-healing assay

Wound-healing assay was performed to assess the migration ability of cancer cells. Briefly, the cells were seeded in a six-well plate the previous night. Once the cell density reached 100% the following day, a scratch was made in the central area using a 1000-µL microgun tip. Subsequently, the medium containing 10% FBS was replaced with a serum-free medium, and the initial scratch area was photographed under a microscope. The cells were then left to culture for 12 h before being photographed again. The ImageJ software was utilized for quantifying the migration region.

### Flow cytometry

Cell apoptosis was assessed using the Annexin V-FITC/PI Apoptosis Detection Kit (Vazyme, China), according to the manufacturer’s instructions. Briefly, 100 µL of a binding buffer was added to the collected cell precipitates and gently mixed. Then, 5 µL of Annexin V-FITC and 5 µL of a propidium iodide staining solution were added, followed by incubation at room temperature for 10 min in a dark room. Next, 400 µL of the binding buffer was added and gently mixed before detection by flow cytometry within 1 h. FlowJo 10.8 software was utilized for the analysis of the apoptosis data.

### Mitochondrial morphology

When the cells in the cell culture dishes reached approximately 30% density, the Mito-Tracker Deep Red FM working solution (Beyotime, China) was added and incubated at 37°C for 30 min. This working solution was then removed and replaced with a Dulbecco’s modified eagle medium containing 10% FBS. The cells were observed using a Zeiss Axio Observer7 inverted fluorescence microscope. The mitochondrial morphology was analyzed using ZEN Microscopy software. Mitochondria with different morphologies (fragmented: length ≤1.0 µm; intermediate: length, 1.0–2.0 µm; elongated: length >2.0 µm) were quantified.^32^ At least 30 cells from each independent experiment were analyzed.

### Tumor models

Male C57BL/6J mice (5-week-old) were provided by Jinan Pengyue Experimental Animal Breeding Co., Ltd. They were fed in a specific pathogen free (SPF) vivarium under standard conditions. Briefly, the mice were fed in a room with a 12-h light – dark cycle under constant temperature (22 °C) and humidity (55%) and were provided with free access to water and food. To construct a subcutaneous tumor model, the mice were randomly placed in two groups: experimental and control, with eight mice in each group. PINCH-1-knockdown and negative control SCC7 cell lines (1 × 10^6^ cells suspended in 100 μL of Matrigel and PBS) were injected into the subcutaneous right axilla of the experimental and control groups, respectively. The tumor was measured using a caliper every three days. The tumor volume was calculated as follows: *a* × *b*^2^/2 (*a*, the largest tumor diameter; *b*, the smallest tumor diameter). The mice were killed by cervical dislocation when the tumor necrosis or volume reached 2000 mm^3^. All processes were performed in an SPF laboratory.

### Bioinformatics analysis

RNA-Seq data (FPKM format) for 28 different tumors, including HNSCC, were downloaded from the Cancer Genome Atlas (TCGA) database (https://portal.gdc. cancer.gov/). The RNA-seq data from the HNSCC samples included data on 484 tumor tissues and 44 normal tissues. We analyzed the expression levels of PINCH-1 in 28 different tumors. R software (version 3.6.4) was utilized for statistical analysis. The visualization was performed using the “ggplot2” (version 3.3.3) package. The Wilcoxon rank sum test was used to analyze the difference in PINCH-1 expression between normal and tumor tissues in each tumor.

### Statistical analysis

GraphPad Prism 8.0 was utilized for data visualization and statistical analysis. A bilateral *p* < .05 was considered to indicate statistically significant results. Student’s *t*-test or one-way ANOVA was used to compare continuous variables. Chi-square/Fisher’s exact tests were applied to compare categorical variables. All experiments were carried out in triplicate unless otherwise specified.

## Results

### PINCH-1 was overexpressed in HNSCC and associated with lymph node metastasis and pathological differentiation grade

**Figure 1.**
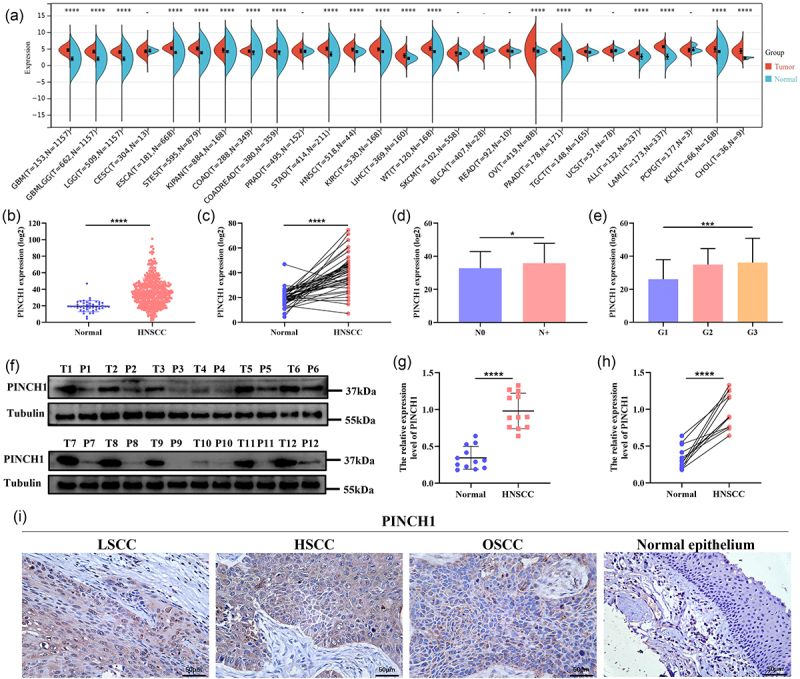
PINCH-1 was highly expressed in HNSCC tissues. (a) mRNA levels of PINCH-1 in pan-cancer from the TCGA database. (b, c) mRNA levels of PINCH-1 in HNSCC tissues from the TCGA database. (d, e) expression level of PINCH-1 was correlated with lymph node metastasis and pathological differentiation grade. (f) Expression level of PINCH-1 protein in HNSCC tissues and para-cancer tissues. (g, h) relative quantitative analysis of PINCH-1 protein in HNSCC and para-cancer tissues. (i) Representative images of immunohistochemical staining of PINCH-1 in HNSCC tissues and normal epithelium tissues at 400× magnification. Scale bar: 50 μm. TCGA, the cancer genome Atlas; T, primary tumor; P, para-cancer; LSCC, laryngeal squamous cell carcinoma; HSCC, hypopharyngeal squamous cell carcinoma; OSCC, oral squamous cell carcinoma. *p < .05, ***p < .001, ****p < .0001.

We initially examined the expression level of PINCH-1 in tumor tissues to investigate its role in HNSCC tumorigenesis. The analysis of RNA-seq data from the TCGA database revealed that PINCH-1 mRNA was overexpressed in various tumors in contrast to that in the corresponding normal tissues (Figure 1(a)). The TCGA database also showed that PINCH-1 mRNA was overexpressed in HNSCC (Figure 1(b,c)). Clinicopathological feature analysis revealed that the high expression of PINCH-1 mRNA was associated with lymph node metastasis and poor pathological differentiation (Figure 1(d,e)). To provide further support, we evaluated the expression level of PINCH-1 protein through Western blotting analysis, which indicated that PINCH-1 was overexpressed in HNSCC (Figure 1(f–h)). Moreover, immunohistochemical analysis showed that the expression level of PINCH-1 protein was elevated in HNSCC tissues compared to that in the adjacent para-cancer tissues (Figure 1i). Notably, an association between the upregulation of PINCH-1 expression and lymph node metastasis was observed through an immunohistochemical staining analysis (Table 1).

**Table 1.** Expression of PINCH1 in HNSCC in relation to clinical features.

Features	Low, *N* (%)	High, *N* (%)	*P*-value
**Age，years**			.741
≤60	7(63.6)	21(56.8)	
>60	4(36.4)	16(43.2)	
**Sex**			
Male	10(90.9)	35(94.6)	.551
female	1(9.1)	2(5.4)	
**Differentiation grade**			
G1/G2	8(72.7)	23(62.2)	.723
G3	3(27.3)	14(37.8)	
**T stage**			
T1/T2	5(45.5)	25(67.6)	.288
T3/T4	6(54.5)	12(32.4)	
**N stage**			
N0	10(90.9)	19(51.4)	.032
N+	1(9.1)	18(48.6)	
**Clinical stage**			
I/II	4(36.4)	11(29.7)	.720
III/IV	7(63.6)	26(70.3)	
**Smoking history**			
Yes	8(72.7)	29(78.4)	.697
No	3(27.3)	8(21.6)	
**Drinking history**			.653
Yes	9(81.8)	32(86.5)	
No	2(18.2)	5(13.5)	

Clinical stage was determined based on 8th AJCC stage.

### PINCH-1 promotes cell proliferation and inhibits cell apoptosis in HNSCC

**Figure 2.**
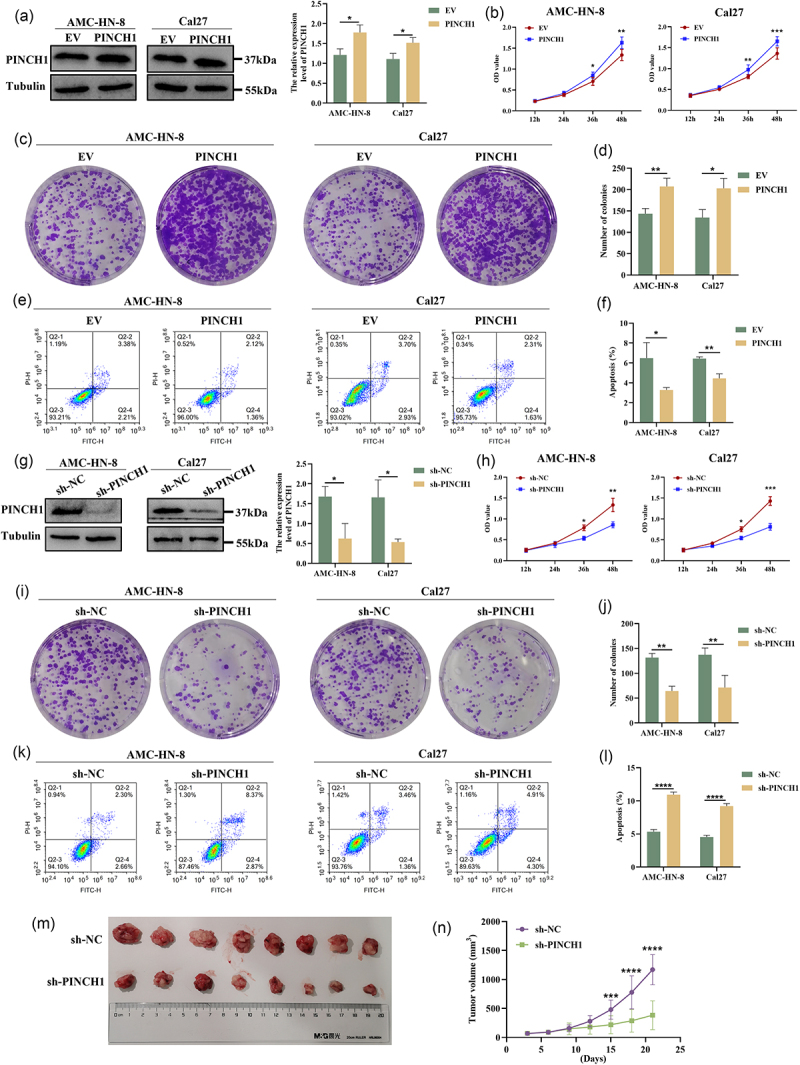
Effect of PINCH-1 on cell proliferation and apoptosis in HNSCC cell lines. (a) Overexpression effect of PINCH-1 in HNSCC cell lines. (b, h) effect of PINCH-1 overexpression on cell proliferation in AMC-HN-8 and Cal27 cell lines. (c, d) effect of PINCH-1 overexpression on cell colony formation in AMC-HN-8 and Cal27 cell lines. (E, F) effect of PINCH-1 overexpression on apoptosis in AMC-HN-8 and Cal27 cell lines. (g) Knockdown effect of PINCH-1 in AMC-HN-8 and Cal27 cell lines. (h) Effect of PINCH-1 knockdown on cell proliferation in HNSCC cell lines. (i,j) effect of PINCH-1 knockdown on cell colony formation in AMC-HN-8 and Cal27 cell lines. (k,l) effect of PINCH-1 knockdown on apoptosis in AMC-HN-8 and Cal27 cell lines. (m.n) effect of PINCH-1 knockdown on tumor growth in vivo. EV, empty vector. *p < .05, **p < .01, ***p < .001, ****p < .0001.

We exogenously upregulated the expression of PINCH-1 in AMC-HN-8 and Cal27 cell lines to explore its potential role in HNSCC (Figure 2(a)). As shown in Figure 2(b), the upregulation of PINCH-1 significantly promoted the proliferation capacity of HNSCC cells. The results of the colony-formation assay further showed that the upregulation of PINCH-1 could markedly enhance the colony-formation capacity of HNSCC cells (Figure 2(c,d)). Additionally, we evaluated the effect of PINCH-1 on HNSCC apoptosis. Flow cytometry apoptosis analysis revealed that the overexpression of PINCH-1 strongly suppressed apoptosis in both AMC-HN-8 and Cal27 cell lines (Figure 2(e,f)). In addition, we downregulated the expression of PINCH-1 using shRNA in HNSCC cell lines (Figure 2(g)). PINCH-1 knockdown not only significantly inhibited the proliferation and colony-formation ability of HNSCC cell lines (Figure 2(h-j)), but also increased the rate of cell apoptosis (Figure 2(k,l)). Next, we investigated the role of PINCH-1 on tumor growth in C57 mice. The downregulation of PINCH-1 expression resulted in a significant inhibition of subcutaneous tumor growth in mice, as evidenced by the tumor growth curve (Figure 2m.n).

### PINCH-1 enhances cell migration and invasion in HNSCC

**Figure 3.**
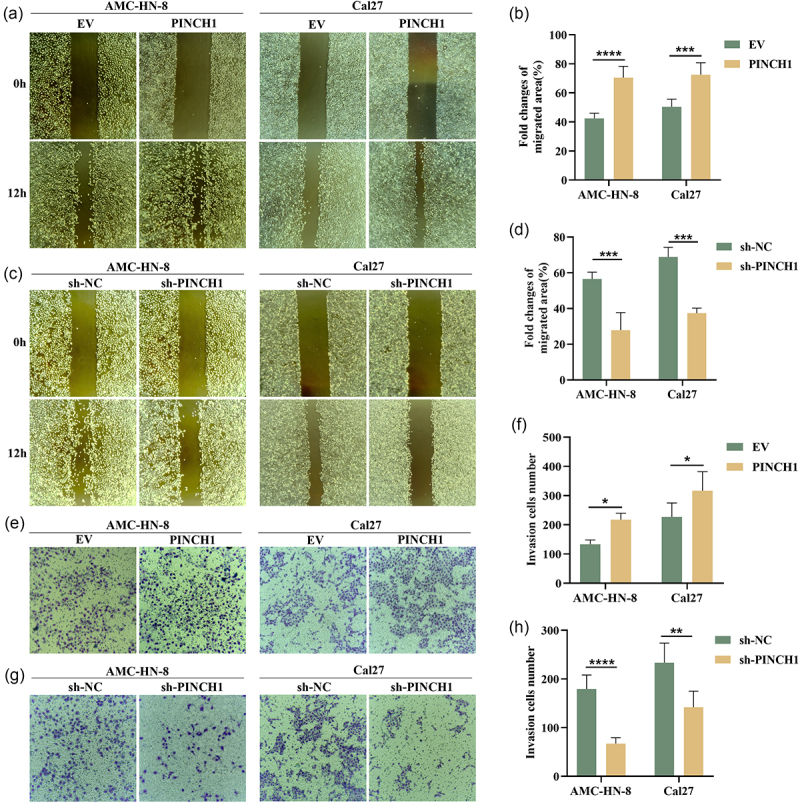
PINCH-1 promotes cell migration and invasion in HNSCC cell lines. (a–d) effect of PINCH-1 overexpression or knockdown on cell migration in AMC-HN-8 and Cal27 cell lines. (e-h) effect of PINCH-1 overexpression or knockdown on cell invasion in AMC-HN-8 and Cal27 cell lines. NC, negative control. EV, empty vector. *p < .05, **p < .01, ***p < .001, ****p < .0001.

Since clinicopathological analysis revealed that the expression level of PINCH-1 is related to lymph node metastasis, we explored the impact of PINCH-1 on the migration and invasion ability of AMC-HN-8 and Cal27 cell lines. Wound-healing assay results showed that the overexpression of PINCH-1 significantly enhanced the migration capacity of the AMC-HN-8 and Cal27 cells (Figure 3(a,b)), whereas its knockdown markedly suppressed cell migration (Figure 3(c,d)). Similarly, the transwell assay revealed that the overexpression of PINCH-1 substantially augmented the invasion capacity of AMC-HN-8 and Cal27 cells (Figure 3(e,f)), while its knockdown markedly restricted cell invasion (Figure 3(g,h)).

### Blocking mitochondrial fission inhibits cell proliferation and metastasis in HNSCC

**Figure 4.**
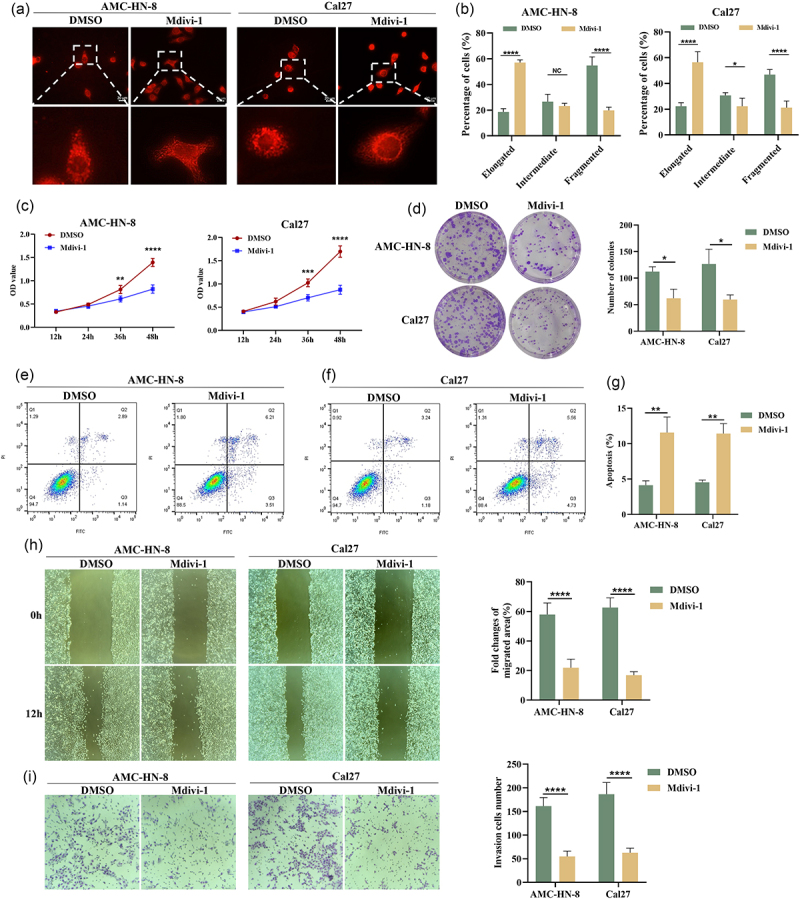
Mitochondrial division inhibitor (Mdivi-1) treatment inhibits the growth and metastasis of HNSCC cell lines. (a) Representative fluorescence images of mitochondrial morphology after Mdivi-1 treatment in AMC-HN-8 and Cal27 cell lines. Scale bar: 20 μm. (b) Quantitative analysis of the proportion of cells with different mitochondrial morphologies after Mdivi-1 treatment. (c) Effect of Mdivi-1 on cell proliferation in AMC-HN-8 and Cal27 cell lines. (d) Effect of Mdivi-1 on colony formation in AMC-HN-8 and Cal27 cell lines. (e-g) effect of Mdivi-1 on cell apoptosis in AMC-HN-8 and Cal27 cell lines. (h) Effect of Mdivi-1 on cell migration in AMC-HN-8 and Cal27 cell lines. (i) Effect of Mdivi-1 on cell invasion in AMC-HN-8 and Cal27 cell lines. DMSO, dimethylsulfoxide. *p < .05, **p < .01, ***p < .001, ****p < .0001.

Mdivi-1 was used to treat HNSCC cell lines to investigate the effects of inhibiting mitochondrial fission on cell function. As shown in Figures 4(a,b), Mdivi-1 inhibited mitochondrial fission in both AMC-HN-8 and Cal27 cell lines, leading to excessive elongation of mitochondria. CCK8 assay results showed that Mdivi-1 inhibited cell proliferation in both AMC-HN-8 and Cal27 cell lines (Figure 4c). Mdivi-1 can also inhibit colony-formation capacity in these two cell lines (Figure 4d). Flow cytometry apoptosis analysis showed that the inhibition of mitochondrial fission could promote apoptosis in AMC-HN-8 and Cal27 cells (Figure 4(e-g)). In addition, wound-healing assay results demonstrated that treatment with Mdivi-1 significantly suppressed the migratory capacity of AMC-HN-8 and Cal27 cells (Figure 4(h)). Similarly, the transwell assay revealed that, following Mdivi-1 treatment, a significant inhibition was observed in cell invasion in AMC-HN-8 and Cal27 cells (Figure 4(i)).

### PINCH-1 regulates DRP1-mediated mitochondrial fission

**Figure 5.**
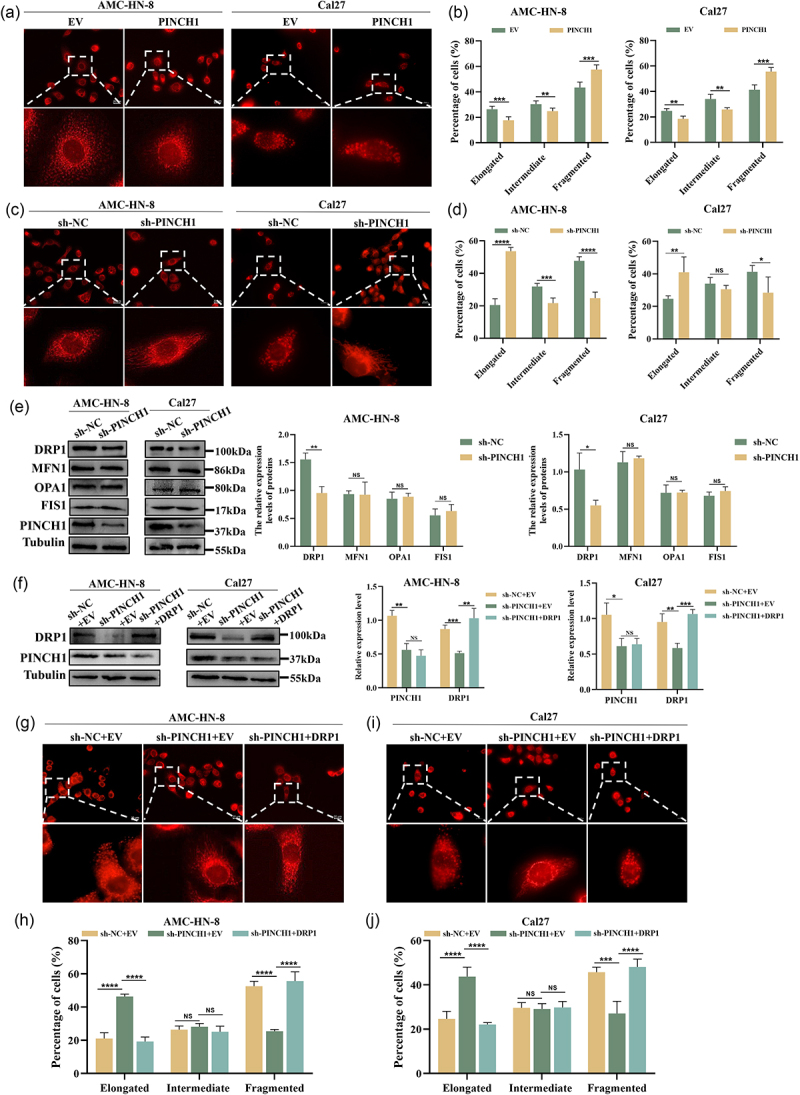
PINCH-1 regulates DRP1-mediated mitochondrial fission in HNSCC cell lines. (a, b) Representative fluorescence images of mitochondrial morphology after PINCH-1 overexpression. Quantification of the percentage of cells with different mitochondrial morphologies. Scale bar: 20 μm. (c, d) Representative fluorescence images of mitochondrial morphology after PINCH-1 knockdown and quantification of the percentage of cells with different mitochondrial morphologies. (e) Effect of PINCH-1 knockdown on mitochondrial dynamics-related protein expression. (f) Overexpression effect of DRP1 in AMC-HN-8 and Cal27 cell lines with PINCH-1 knockdown. (g, i) Representative fluorescence images of mitochondrial morphology after DRP1 overexpression in PINCH-1 knockdown cells. (h, j) quantitative analysis of the proportion of cells with different mitochondrial morphologies. NC, negative control. EV, empty vector. *p < .05, **p < .01, ***p < .001, ****p < .0001.

As depicted in Figure 5a, the overexpression of PINCH-1 enhanced mitochondrial fission and induced mitochondrial fragmentation. Compared to the control group, the PINCH-1-overexpression group had a higher proportion of cells with fragmented mitochondria and a lower proportion of cells with elongated mitochondria (Figure 5(b)). Conversely, the downregulation of PINCH-1 expression in AMC-HN-8 and Cal27 cell lines promoted mitochondrial fusion, resulting in an excessive elongation of the mitochondria (Figure 5(c,d)). To further elucidate the regulatory mechanism of PINCH-1 on mitochondrial dynamics, we examined key proteins involved in this process. The results demonstrated a significant decrease in DRP1 expression following the downregulation of PINCH-1 in both AMC-HN-8 and Cal27 cell lines, while the expression levels of MFN1, OPA1, and FIS1 remained unchanged (Figure 5(e)). Subsequently, we employed lentiviral transfection to upregulate DRP1 expression in AMC-HN-8 and Cal27 cells with PINCH-1 knockdown (Figure 5(f)). As depicted in Figures 5(g,h), the upregulation of DRP1 in AMC-HN-8 cells counteracted the mitochondrial fusion induced by PINCH-1 knockdown and reinstated mitochondrial fission. In addition, we observed that the re-expression of DRP1 reversed the mitochondrial fusion induced by PINCH-1 knockdown in Cal27 cells (Figure 5(i,j)).

### PINCH-1 promotes the growth and metastasis of HNSCC by modulating DRP1-mediated mitochondrial fission

**Figure 6.**
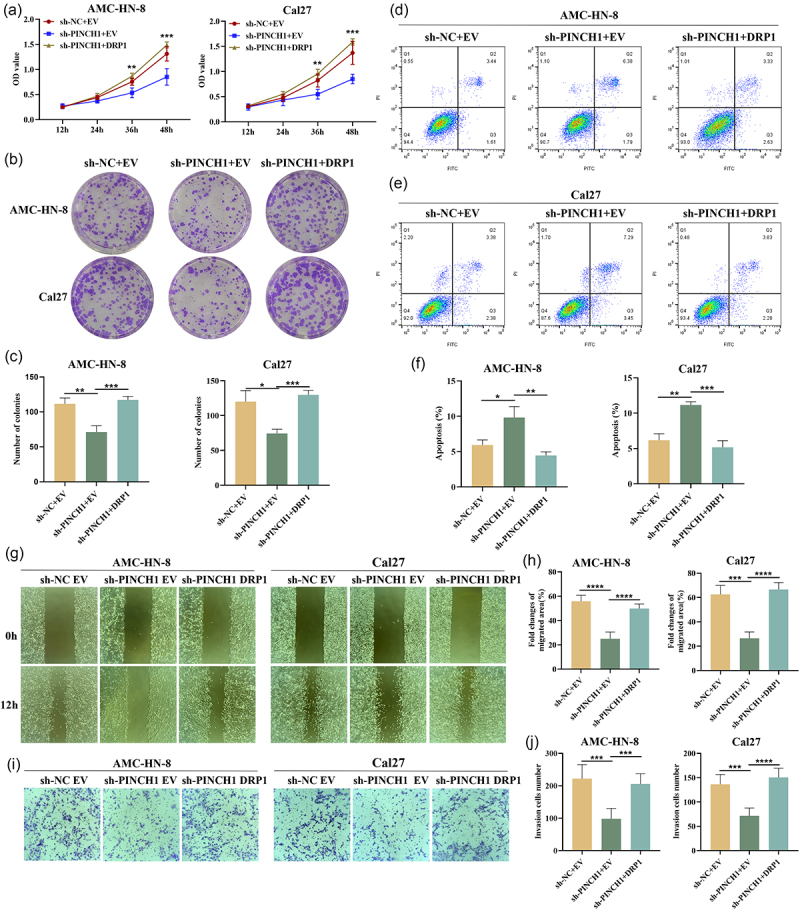
Overexpression of DRP1 reversed the effects of PINCH-1 knockdown on HNSCC cell lines. (a) DRP1 overexpression counteracted the decrease in cell proliferation induced by PINCH-1 knockdown in AMC-HN-8 and Cal27 cell lines. (b, c) overexpression of DRP1 in PINCH-1 knockdown AMC-HN-8 and Cal27 cell lines restored the colony-formation capacity. (d-f) apoptosis induced by PINCH-1 knockdown in AMC-HN-8 and Cal27 cell lines was reversed with DRP1 overexpression. (g, h) DRP1 overexpression reversed the impaired migration ability resulting from PINCH-1 knockdown in AMC-HN-8 and Cal27 cell lines. (i, j) overexpression of DRP1 in PINCH-1 knockdown restored the invasive capacity of AMC-HN-8 and Cal27 cell lines. NC, negative control. EV, empty vector. *p < .05, **p < .01, ***p < .001, ****p < .0001.

Cell function experiments demonstrated that exogenously upregulating DRP1 expression effectively counteracted the decrease in cell proliferation induced by PINCH-1 downregulation in AMC-HN-8 and Cal27 cell lines (Figure 6(a)). Colony-formation assay results also demonstrated that the re-expression of DRP1 in PINCH-1-knockdown AMC-HN-8 and Cal27 cells restored the colony-formation capacity (Figure 6(b,c)). Furthermore, flow cytometry analysis revealed that apoptosis promotion induced by PINCH-1 downregulation in AMC-HN-8 and Cal27 cell lines reversed upon the upregulation of DRP1 expression (Figure 6(d-f)). As shown in Figures 6(g,h), re-expression of DRP1 in AMC-HN-8 and Cal27 cells reversed the impaired migration ability resulting from PINCH-1 knockdown. In addition, transwell experiments showed that the upregulation of DRP1 expression in AMC-HN-8 and Cal27 cells with PINCH-1 knockdown restored their invasive capacity (Figure 6(i,j)).

## Discussion

PINCH-1 was overexpressed in the HNSCC epithelium in contrast to that in the normal mucosal epithelium. Additionally, the expression level of PINCH-1 was correlated with lymph node metastasis and poor pathological differentiation, suggesting that PINCH-1 might be involved in the development and metastasis of HNSCC. Our research further showed that PINCH-1 promotes HNSCC growth and metastasis by driving DRP1-mediated mitochondrial fission. These findings reveal a novel biological mechanism that regulates the occurrence and progression of HNSCC.

PINCH-1 is an adaptor protein that interacts with several binding partners, activating downstream signaling pathways crucial for cell proliferation, survival, and motility.^25^ Elevated expression of PINCH-1 has been reported in various cancer types, indicating its association with the promotion of tumorigenesis.^27,29,30^ However, the biological role of PINCH-1 in HNSCC is yet to be explored. A previous study revealed that PINCH-1 is overexpressed in laryngeal squamous cell carcinoma (LSCC), and that its expression level is correlated with lymph node metastasis and poor pathological differentiation.^33^ Our study further demonstrated that PINCH-1 is not only overexpressed in LSCC, but also in other HNSCC types, including hypopharyngeal and oropharyngeal squamous cell carcinomas. In addition, we observed that the high expression of PINCH-1 is associated with lymph node metastasis and pathological differentiation. Our results suggest that PINCH-1 may play an important role in promoting the initiation and progression of HNSCC. Previous studies have demonstrated that PINCH-1 can promote proliferation, inhibit apoptosis, enhance metastasis, and resist radiotherapy in malignant tumors.^25,29,34^ These findings were essentially consistent with those of our current study. We observed that the overexpression of PINCH-1 in HNSCC cells promotes cell proliferation, migration, and invasion. Hence, it can be suggested that targeting dysregulated PINCH-1 may be employed as a novel strategy for suppressing HNSCC growth and metastasis.

Mitochondrial dynamics, characterized by the continuous fusion and fission of mitochondria, is considered a crucial biological process in carcinogenesis.^32^ There is increasing evidence to suggest that tumor cells gain proliferative and survival advantages by disrupting the dynamic balance between mitochondrial fusion and fission.^35^ Enhanced mitochondrial fission has been observed in various tumors, such as colon cancer, gastric cancer, and esophageal cancer.^19,36,37^ Notably, changes in mitochondrial dynamics are mainly regulated by several key proteins, including DRP1, FIS1, MFN1, and OPA1.^35^ DRP1, the critical GTPase for mitochondrial fission, is upregulated in many cancers and is closely implicated in tumorigenesis.^19^ Huang et al. showed that DRP1 is overexpressed in HNSCC and closely associated with a poor prognosis.^21^ Although they showed that a high expression of DRP1 promotes the initiation and progression of HNSCC, the upstream regulatory mechanism has not yet been elucidated. In the current study, we showed that PINCH-1 can act as an upstream regulator and enhance DRP1-mediated mitochondrial fission. As an important component of the integrin adhesion complex, PINCH-1 participates in regulating cell – ECM interactions and signal transduction, thereby playing a crucial role in promoting tumor progression and metastasis.^25^ Previous studies have implicated cell – ECM adhesion in the regulation of mitochondrial dynamics.^22^ Furthermore, the PINCH/ILK/parvin complex works collaboratively with transmembrane integrins and growth factor receptors through the PI3K/PKB/Akt1 and Ras/MAPK cascades to enable synergistic signaling to regulate downstream functional proteins.^38^ Therefore, our finding that PINCH-1 regulates DRP1-mediated mitochondrial fission is convincing. However, the specific signaling pathway between PINCH1 and DRP1 remains to be explored. In addition, given the facilitative role of PINCH-1 in HNSCC growth and metastasis, exploring whether it drives HNSCC progression by modulating mitochondrial dynamics is a critical step in understanding its role in tumorigenesis.

Our investigation showed that PINCH-1 promotes cell proliferation and inhibits apoptosis by regulating DRP1-mediated mitochondrial fission. One of the key features of cancer cells is their ability to deregulate cell cycle checkpoints, which allows them to proliferate indefinitely. Mitochondrial fission plays a major role in various stages of the cell cycle, which is crucial for the smooth progression of cell division.^7,39^ However, excessive mitochondrial fission is associated with the promotion of cell proliferation in malignant tumors, such as liver, stomach, and esophageal cancers.^19,37,40^ A previous study demonstrated that DRP1-mediated mitochondrial fission is associated with the G1–S phase transition of hepatocellular cancer (HCC) cells, which accelerates cell proliferation by promoting cell cycle progression.^41^ Therefore, we speculate that the role of PINCH-1 in promoting cell proliferation in HNSCC may be related to the regulation of the cell cycle through DRP1-mediated mitochondrial fission. The ability to evade apoptosis is also an important feature of cancer cells. Changes in the expression of regulatory proteins for mitochondrial dynamics promote this phenotype.^7^ Huang et al. demonstrated that DRP1-mediated mitochondrial fission enhances the survival of HCC cells by suppressing mitochondria-dependent apoptosis.^40^ They reported that the overexpression of DRP1 inhibited the release of cytochrome c as well as the cleavage of caspases 9 and 3. In colon cancer and thyroid cancer, the researchers also found that DRP1 can enhance the anti-apoptotic ability of cells by interfering with the release of apoptotic proteins.^42,43^ This could be a possible mechanism through which DRP1-mediated mitochondrial fission protects HNSCC cells against apoptosis.

Furthermore, mitochondrial fission is believed to be involved in the regulation of tumor metastasis. Yan et al. observed that the binding of Circ_0098823 to IGF2BP3 regulates the expression of DRP1, thereby promoting HCC metastasis through mitochondrial fission.^44^ Xie et al. demonstrated that STMP1 promotes tumor metastasis by enhancing DRP1-mediated mitochondrial fission.^15^ We similarly identified a pro-carcinogenic role of PINCH-1 in enhancing the migration and invasion of HNSCC by promoting DRP1-mediated mitochondrial fission. Furthermore, it has been reported that mitochondria are fragmented in a DRP1-dependent manner for transportation and accumulation at the leading edge of invasive breast cancer cells, where they promote the production of local ATP essential for the formation of lamellipodia and for cell metastasis.^45^ The same phenomenon was observed in lung cancer, epithelial cancer, and thyroid cancer.^13,46,47^ These findings provide a reference for further understanding of the mechanism through which DRP1-mediated mitochondrial fission promotes metastasis in HNSCC.

In summary, our study showed that PINCH-1 is overexpressed in HNSCC tissues and promotes the growth and metastasis of HNSCC by driving DRP1-mediated mitochondrial fission. Because modulation of mitochondrial dynamics is attracting attention as one of the crucial strategies of anti-tumor therapy, our work identifies new intervention targets for HNSCC treatment. Future studies on the topic should explore the signaling pathway of PINCH-1 upregulating DRP1 expression and the potential mechanism of mitochondrial fission for regulating cell proliferation and metastasis.

## Data Availability

The bioinformatics analysis datasets during the current study are available in the TCGA database (https://portal.gdc. cancer.gov/). All the data and material in this paper are available when requested.

## Abbreviations

DRP1: dynamin-related protein 1
ECM: extracellular matrix
FBS: fetal bovine serum
HCC: hepatocellular cancer
HNSCC: head and neck squamous cell carcinoma
IHC: immunohistochemistry
LSCC: laryngeal squamous cell carcinoma
Mdivi-1: mitochondrial division inhibitor
PBS: phosphate-buffered saline
PINCH-1: particularly interesting new cysteine-histidine rich protein 1
PVDF: polyvinylidene fluoride
shRNA: short-hairpin RNA
SPF: specific pathogen free
